# Characteristic Renal Histology of a 81-Year-Old Patient with a 30-Year History of Diabetes Mellitus: A Case Report

**DOI:** 10.1007/s13730-020-00483-9

**Published:** 2020-05-02

**Authors:** Natsuki Shima, Naoki Sawa, Masayuki Yamanouchi, Hiroki Mizuno, Masahiro Kawada, Akinari Sekine, Rikako Hiramatsu, Noriko Hayami, Eiko Hasegawa, Tatsuya Suwabe, Junichi Hoshino, Kenmei Takaichi, Kenichi Ohashi, Takeshi Fujii, Yoshifumi Ubara

**Affiliations:** 1 Nephrology Center, Toranomon Hospital, 1-3-1, Takatsu, KajigayaKawasaki, Kanagawa 212-0015 Japan; 2 Okinaka Memorial Institute for Medical Research, Toranomon Hospital, Tokyo, Japan; 3 Department of Pathology, Toranomon Hospital, Tokyo, Japan; 4 Department of Pathology, Yokohama City University Hospital Graduate School of Medicine, Kanagawa, Japan; 5 Division of Rheumatology and Clinical Immunology, Department of Medicine, Jichi Medical University, Tochigi, Japan

**Keywords:** Diabetic kidney disease, Diabetic nephropathy, Hyperfiltration, Hyperperfusion, Polar vasculosis

## Abstract

A renal histology of an 81-year-old man with a 30-year history of diabetes mellitus (DM), as well as diabetic retinopathy and neuropathy, was examined. The patient’s blood pressure was controlled within the normal range (less than 140/75 mmHg) using antihypertensive agents including angiotensin receptor blocker. Edematous management was achieved by a strict salt diet (less than 6 g/per day). However, this patient’s glycemic control was poor with HbA1c 8–10%. Serum creatinine was 0.87 mg/dL and estimated globular filtration rate (eGFR) was 64 ml/min/1.73m^2^. Urinary protein excretion was 1.5 g/day. This patient’s renal biopsy showed linear staining for IgG along the GBM by immunofluorescence microscopy, but light microscopy showed almost intact glomeruli, and the GBM was not thickened as revealed by electron microscopy with a width of 288–368 nm (< 430 nm). While arteriolar hyalinosis was severe, and polar vasculosis was observed around the glomerular vascular pole. This case indicates that long-standing hyperglycemia may induce polar vasculosis by the mechanism of angiogenesis, but diabetic glomerulopathy can become minor change, only when hypertension and edematous management could be controlled strictly.

## Introduction

Diabetic kidney disease (DKD) develops in approximately 40% of diabetic patients and is the leading cause of chronic kidney disease (CKD) worldwide. If complete diabetic management is not in place, diabetic nephropathy (DN) may occur. Diabetic nephropathy is characterized by typical lesions such as nodular lesions, or exudative lesions resulting ultimately in endo-stage renal disease [1, 2]. Mise et al. reported the renal biopsies of 205 patients with diabetes and overt proteinuria, and various types of histologies were noted. According to the Tervaert’s classification, typical nodular lesions (class III) were noted on 57(28%) out of 205 patients, and mild type (class 1 and IIA) was seen in 57 patients (28%) [3]. Yamanouchi et al. reported the existence of non-proteinuric type in CKD stage III patients with DKD [4].

Several small vessels were frequently observed around the glomerular vascular pole in DN, known as polar vasculosis; however, why this occurs remains unclear [5]. In this case study, polar vasculosis was investigated in an 81-year-old man with a long-term diabetic history.

## Case Report

**Fig. 1:**
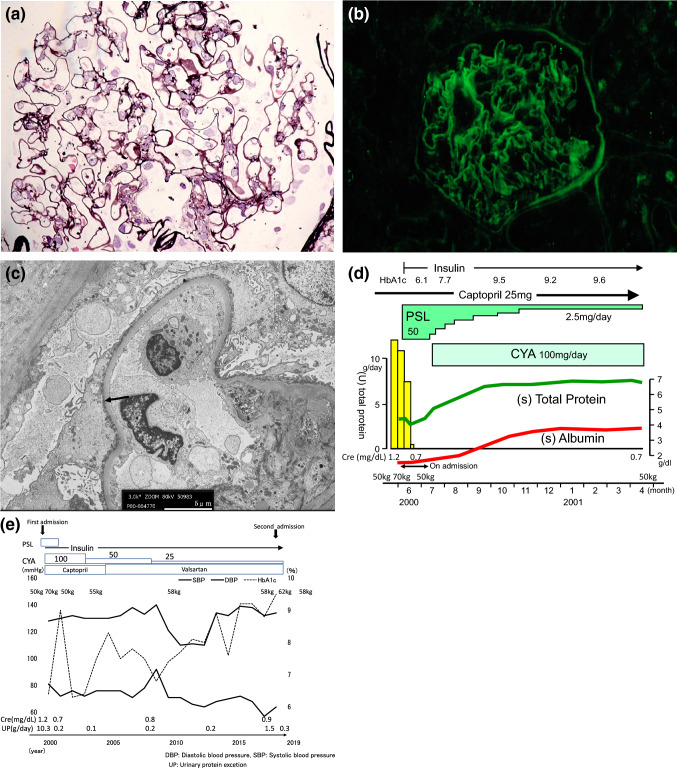
**a** LM revealed intact glomeruli. **b** Immunofluorescence (IF) revealed linear immunofluorescent staining for IgG along the glomerular basement membranes (GBM). **c** electron microscopy (EM) revealed foot process effacement (arrow). The GBM was not thickened as revealed by EM with a width of 220–250 nm. **d** Clinical course on first admission. **e** All clinical course

An 81-year-old Japanese man was admitted to the authors’ hospital for evaluation of a weight gain of 4.0 kg and the exacerbation of proteinuria up to 1.5 g daily. Hypertension was noted at the age of 46. Diabetes mellitus (DM) was diagnosed at the age of 51. Oral hypoglycemic drugs were started, but his glycemic control was poor with HbA1c 8–10%. This patient’s body weight was increased from 50 to 70 kg at the interval of 1 month at the age of 63. Proteinuria in the nephrotic range with 10.3 g daily developed. The first renal biopsy was as follows: global sclerosis as observed via light microscopy (LM), was present in 1 out of 15 glomeruli. LM revealed intact glomeruli (Fig. 1a); immunofluorescence (IF) revealed linear immunofluorescent staining for IgG along the glomerular basement membranes (GBM) (Fig. 1b), and electron microscopy (EM) revealed foot process effacement (Fig. 1c). The GBM was not thickened with a width of 220–250 nm. Arteriolar hyalinosis was mild, and mild polar vasculosis was seen around the glomeruli. Minimal change nephrotic syndrome was diagnosed. Prednisolone therapy of 50 mg daily was started, and cyclosporine of 100 mg daily was added and complete remission achieved, and this patient’s body weight was decreased from 70 to 50 kg (Fig. 1d). Prednisolone was discontinued after 1 year, but cyclosporine of 25 mg daily was continued. With the start of this treatment, intensive insulin therapy was started, but following this, for approximately 18 years, hypoglycemic attack was easy to be caused and his glycemic control was HbA1c 8–10%. The patient’s blood pressure was controlled within the normal range (less than 140/75 mmHg) using antihypertensive agents including angiotensin receptor blocker (Fig. 1e). Edematous management was achieved by a strict salt diet (less than 6 g/per day). A month before hospital admission, the patient began to overeat and overdrink resulting in a weight gain of 4 kg and leg edema was noted.

On admission, the patient was 158 cm tall and weighed 62 kg, with a blood pressure of 138/68 mmHg, a heart rate of 88 /min, and a temperature of 36.2 °C. Edema was noted on the lower extremities. This patient showed mild to moderate nonproliferative diabetic retinopathy that does not need treatment, and peripheral neuropathy including numbness and burning sensation, but clinical sign of coronary diseases and obstructive arteriosclerosis was not detected. Smoking history and family history of diabetes were not noted.

Laboratory tests revealed a leukocyte count of 5600/μL, hemoglobin was 12.4 g/dL, and a platelet count of 28.3 × 10^4^/μL. Serum albumin was 4.1 g/dL, blood urea nitrogen was 15 mg/dL, creatinine was 0.87 mg/dL, and estimated globular filtration rate (eGFR) was 64 ml/min/1.73m^2^. Plasma glucose was 154 mg/dL and HbA1c levels were 9.9%. Total cholesterol was 167 mg/dL and triglyceride was 65 mg/dL. Urinary protein excretion was 1.5 g/day and the urinary sediment contained no erythrocytes or casts. Chest radiograph was normal.

## Repeated Renal Biopsy Findings

**Fig. 2:**
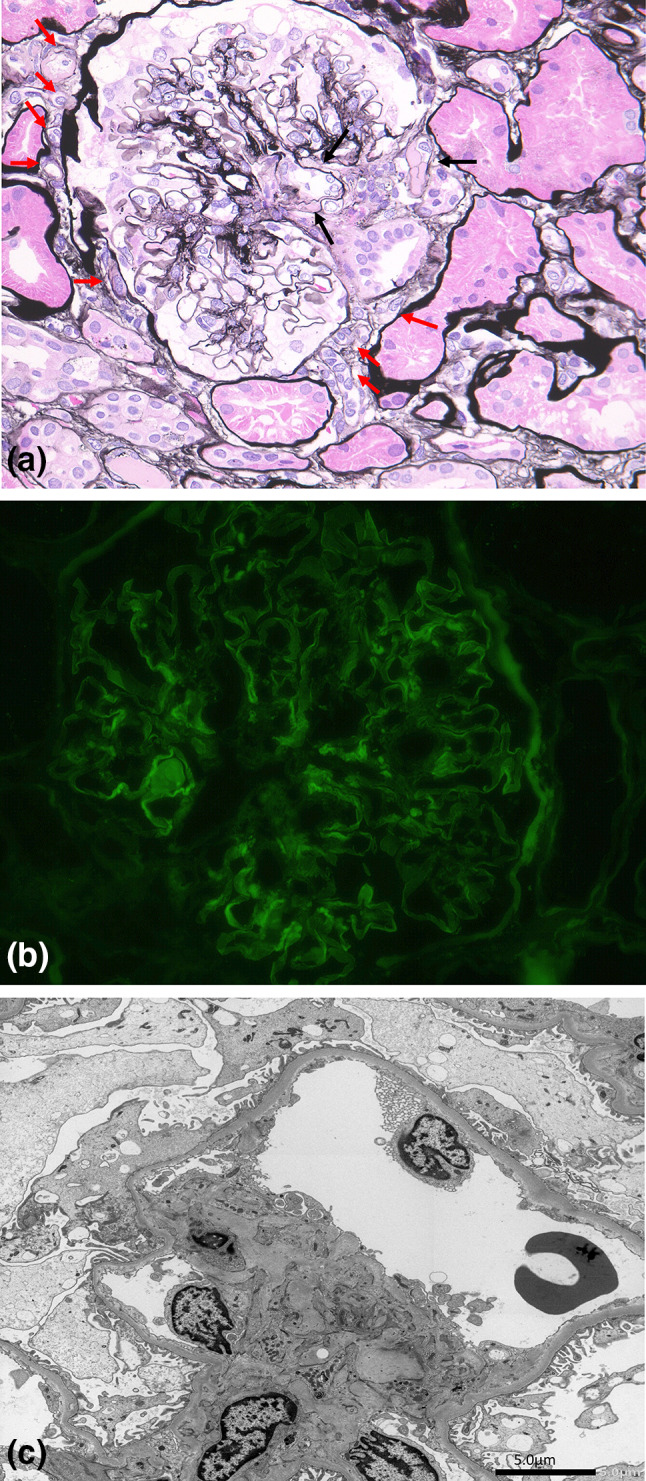
**a** Arteriolar hyalinosis (black arrow), and massive polar vasculosis (small vessels, or neovascularization) (red arrow) around the glomeruli including glomerular pole. **b** immunofluorescence showing linear staining for IgG along the GBM. **c** Electron microscopy revealed no thickening of GBM with a width of 280–420 nm (< 430 nm), and no foot process fusion

Global sclerosis as observed via LM, was present in 4 out of 13 glomeruli. The preserved glomeruli were mostly intact, and there was no evidence of nodular lesions, or mesangial expansion. Arteriolar hyalinosis, however, was severe, and massive polar vasculosis was seen around the glomeruli (Fig. 2a). Tubular atrophy and tubulointerstitial fibrosis occupied less than 25% of the total renal cortex. Linear staining for IgG along the GBM was observed by IF (Fig. 2b). The GBM became thickened by EM with a width of 280–420 nm (< 430 nm), compared with the first biopsy, and foot process fusion was not noted (Fig. 2c). This patient’s renal histology can be diagnosed diabetic nephropathy because of linear staining for IgG along the GBM that is characteristic to DM nephropathy, but the glomerular lesion was minor, and does not arrive at the early stage (class 1) of Tervaert’s pathologic classification of diabetic nephropathy, though the GBM became thickened compared with the first biopsy. While massive polar vasculosis with arteriolar hyalinosis was characteristic.

## Clinical Course

A strict salt diet (less than 6 g daily) was restarted and patient’s weight decreased to 58 kg with a loss of 4 kg per week. The patient’s proteinuria decreased to less than 0.3 g daily.

## Discussion

Min et al. performed a three-dimensional analysis of increased vasculature around the glomerular vascular pole in patients with DN. These vasculatures included the afferent arteriole, anastomosed capillary, and the long growing and well-developing efferent arteriole running into the peritubular capillary around the proximal tubule [5].

Hyperglycemia, overhydration, and hypertension due to excessive salt intake have been considered to induce glomerular change including mesangial expansion and nodular lesion, specific to DN, via glomerular hyperfiltration or hyperperfusion [1]. However, the underlying mechanisms of glomerular hyperfiltration in diabetes remain unclear [6]. One plausible mechanism is increased proximal tubular reabsorption of glucose via sodium–glucose cotransporter 2, which decreases distal delivery of sodium chloride to macula densa, resulting in dilation of the afferent arteriole. This ultimately leads to an increase in glomerular perfusion and results in high, local production, of angiotensin II at the efferent arteriole that leads to vasoconstriction [7]. This patient had a history of hypertension and had taken angiotensin receptor blocker. It is possible that the angiotensin receptor blocker and edematous management by a strict salt diet inhibited the production of angiotensin II at the efferent arteriole and, therefore, prevented glomerular hyperfiltration and progression of DKD, despite the duration of DM for 30 years.

Polar vasculosis, perihilar neovascularization, is abnormal angiogenesis observed around the glomerular vascular pole in DN [8, 9]. A review article suggested that hyperglycemia or hypoxia, promotes vascular endothelial growth factor (VEGF) production, which stimulates endothelial cell proliferation by the mechanism of angiogenesis [10].

Tervaert et al. divided diabetic nephropathy (glomerulopathy) into four hierarchical glomerular lesions from the viewpoint of glomerular lesion using LM and EM. Class 1 was early stage and was defined mild or nonspecific LM changes and EM-proven GBM thickening [11]. On this patient, the first renal biopsy specimen that was performed because of minimal change nephrotic syndrome showed very early stage of diabetic nephropathy revealing only linear immunofluorescent staining for IgG along GBM. Arteriolar hyalinosis was mild, and mild polar vasculosis was seen around the glomeruli. Second renal biopsy also showed that glomerular lesion remained minor, but massive polar vasculosis with arteriolar hyalinosis became definite. This case indicates that diabetic nephropathy includes two characteristics of diabetic vasculopathy and diabetic glomerulopathy. On this patient, diabetic vasculopathy including polar vasculosis and arteriolar hyalinosis became prominent, and diabetic glomerulopathy was mild.

**Fig. 3:**
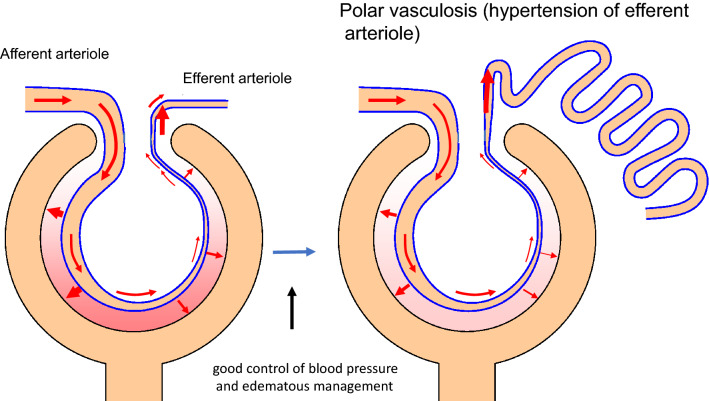
Shema of polar vasculosis: a good control of blood pressure and edematous management can prevent mesangial expansion and nodular lesion via glomerular hyperfiltration or hyperperfusion. This results in a restriction of an overflow of blood into the glomeruli. Simultaneously, an increase in the inflow from the afferent arteriole to the efferent arteriole may result in polar vasculosis, and decrease diabetic glomerular damage

Hyperglycemia for a long period of time has been considered to promote angiogenesis. When overhydration and hypertension were loaded upon hyperglycemia, glomerular damage including mesangial expansion and nodular lesion could be induced by the mechanism of hyperfiltration or hyper perfusion by loading over blood flow into the glomeruli, as well as by the mechanism of angiogenesis. While, when overhydration and hypertension were controlled strictly using antihypertensive agents including angiotensin receptor blocker and a salt diet, glomerular damage via the above hemodynamics become minimum, but continuation of hyperglycemia could be considered to develop proliferation and elongation of efferent arteriole by the mechanism of angiogenesis, and increases the inflow from the afferent arteriole, resulting in polar vasculosis or peri-hilar neovascularization (Fig. 3).

## Abbreviations

CKD: Chronic kidney disease
DKD: Diabetic kidney disease
DM: Diabetes mellitus
DN: Diabetic nephropathy
eGFR: Estimated globular filtration rate
EM: Electron microscopy
GBM: Glomerular basement membrane
HbA1c: Glycated hemoglobin
IF: Immunofluorescence
IgG: Immunoglobulin G
LM: Light microscopy
VEGF: Vascular endothelial growth factor

## References

[1] Alicic RZ, Rooney MT, Tuttle KR (2017). Diabetic kidney disease: challenges, progress, and possibilities. Clin J Am Soc Nephrol.

[2] Wada T, Shimizu M, Yokoyama H, Iwata Y, Sakai Y, Kaneko S (2013). Nodular lesions and mesangiolysis in diabetic nephropathy. Clin Exp Nephrol.

[3] Mise K, Hoshino J, Ubara Y, Sumida K, Hiramatsu R, Hasegawa E, Yamanouchi M, Hayami N, Suwabe T, Sawa N, Fujii T, Ohashi K, Hara S, Takaichi K (2014). Renal prognosis a long time after renal biopsy on patients with diabetic nephropathy. Nephrol Dial Transplant..

[4] Yamanouchi M, Furuichi K, Hoshino J, Toyama T, Hara A, Shimizu M, Kinowaki K, Fujii T, Ohashi K, Yuzawa Y, Kitamura H, Suzuki Y, Sato H, Uesugi N, Hisano S, Ueda Y, Nishi S, Yokoyama H, Nishino T, Samejima K, Kohagura K, Shibagaki Y, Mise K, Makino H, Matsuo S, Ubara Y, Wada T, Research Group of Diabetic Nephropathy, The Ministry of Health, Labour and Welfare, The Japan Agency for Medical Research and Development (2019). Nonproteinuric versus proteinuric phenotypes in diabetic kidney disease: a propensity score-matched analysis of a nationwide, biopsy-based cohort study. Diabetes Care.

[5] Min W, Yamanaka N (1993). Three-dimensional analysis of increased vasculature around the glomerular vascular pole in diabetic nephropathy. Virchows Arch A Pathol Anat Histopathol.

[6] Premaratne E, Verma S, Ekinci EI, Theverkalam G, Jerums G, MacIsaac RJ (2015). The impact of hyperfiltration on the diabetic kidney. Diabetes Metab.

[7] Heerspink HJ, Perkins BA, Fitchett DH, Husain M, Cherney DZ (2016). Sodium glucose cotransporter 2 inhibitors in the treatment of diabetes mellitus: cardiovascular and kidney effects, potential mechanisms, and clinical applications. Circulation.

[8] Furuichi K, Yuzawa Y, Shimizu M, Hara A, Toyama T, Kitamura H (2018). Nationwide multicentre kidney biopsy study of Japanese patients with type 2 diabetes. Nephrol Dial Transplant.

[9] Kanesaki Y, Suzuki D, Uehara G, Toyoda M, Katoh T, Sakai H (2005). Vascular endothelial growth factor gene expression is correlated with glomerular neovascularization in human diabetic nephropathy. Am J Kidney Dis.

[10] Yu CG, Zhang N, Yuan SS, Ma Y, Yang LY, Feng YM (2016). Endothelial progenitor cells in diabetic microvascular complications: friends or foes?. Stem Cells Int.

[11] Tervaert TW, Mooyaart AL, Amann K, Cohen AH, Cook HT, Drachenberg CB, Ferrario F, Fogo AB, Haas M, de Heer E, Joh K, Noël LH, Radhakrishnan J, Seshan SV, Bajema IM, Bruijn JA, Renal Pathology Society (2010). Pathologic classification of diabetic nephropathy. J Am Soc Nephrol..

